# Acupuncture in musculoskeletal pain: analysis of changes in pain perception using the NRS (Numeric Rating Scale)

**DOI:** 10.3389/fpain.2023.1294428

**Published:** 2024-01-08

**Authors:** Lorenzo Fracchia, Alda Maria Olivero, Riccardo Rustichelli, Tiziana Pedrali

**Affiliations:** Center for Studies on Natural and Physical Therapies (CSTNF), Turin, Italy

**Keywords:** acupuncture, musculoskeletal pain, complementary medicine, pain reduction, numeric rating scale, musculoskeletal conditions

## Abstract

**Objectives:**

This study is based on data collected at the Acupuncture clinic of the Local Health Authority (ASL) in Turin from 2008 to 2022 and aims at evaluating the effectiveness of acupuncture in the treatment of musculoskeletal pain using the Numeric Rating Scale (NRS) which analyzes changes in pain perceived by patients in different body regions.

**Methods:**

The database consists of data provided by patients during the initial visit and the last session. Only patients who provided data at the beginning and end of treatment were included. The data were processed using JASP 0.17.2.1 software. The sample consisted of 932 patients with musculoskeletal conditions, excluding 254 subjects with internal medical conditions, who were treated during the same period. The selected population includes individuals aged 23–94, comprising 242 men and 690 women. Patients followed a therapeutic protocol based on the initial diagnosis and underwent an initial cycle of six weekly sessions, with the possibility of four additional sessions if needed. Acupuncture was performed by experienced medical personnel following Traditional Chinese Medicine guideline.

**Results:**

The average NRS values were 7.49 at the beginning and 4.27 at the end, with a 43% reduction in pain. The data were analyzed using the Wilcoxon test, confirming statistical significance (*p* < 0.001). They were then divided by body region, showing a reduction in pain ranging from 40% to 55%. Statistical analysis among different conditions was performed using the Kruskal–Wallis test, with further comparisons using the Dunn test.

**Discussion:**

The study demonstrates that acupuncture is effective in reducing musculoskeletal suffering, with a significant decrease in pain perceived by patients. The results suggest that acupuncture can be a valid treatment for a wide range of conditions, with pain reduction ranging from 40% to 55% and greater effectiveness for elbow-related conditions. However, it is important to note that sample size may influence the results, and further research is needed to confirm and expand these findings, especially for less-represented conditions by the sample.

## 1 Introduction

Musculoskeletal pain represents one of the most common and debilitating clinical issues in contemporary medicine. Its manifestation can range from mild discomfort to a debilitating sensation that significantly impacts the quality of life of patients. Despite advances in pharmacotherapy and physical therapies, the effective treatment of musculoskeletal pain remains an area of research and clinical practice in continuous evolution.

The impact of musculoskeletal conditions on individuals can be estimated through various indices, with the most commonly used being the Disability-Adjusted Life Year (DALY), which corresponds to the sum of years of life lost to premature mortality (Years of Life Lost—YLLs) and years of life lived in suboptimal health or disability (Years of Life lived with Disability—YLDs) due to a specific condition. Musculoskeletal conditions and the resulting pain represent the fourth leading cause of disability (calculated via DALY index) in the elderly, following cardiovascular diseases, neoplasms, and chronic respiratory diseases (1).

Acupuncture, a millennia-old practice integral part to Traditional Chinese Medicine, has garnered increasing interest in the Western scientific and medical community as a possible therapeutic approach for managing musculoskeletal pain by stimulating specific points on the body through the insertion of thin needles. While the origins of acupuncture are rooted in holistic philosophy, its efficacy in treating musculoskeletal pain has sparked intense interest and growing scientific research.

In recent decades, numerous studies have examined the effectiveness of acupuncture in reducing musculoskeletal pain, providing a body of evidence supporting its clinical use. A systematic review by Vickers et al. (2) assessed the effect of acupuncture on chronic pain treatment and reported promising results in reducing musculoskeletal pain and fatigue. Even earlier, a study by Tough et al. (3) demonstrated how acupuncture could positively influence levels of neurotransmitters involved in pain modulation, such as endorphins and serotonin, contributing to its analgesic efficacy.

Understanding the underlying mechanisms of acupuncture's effectiveness in treating musculoskeletal pain is continually evolving: recent research has suggested that acupuncture may influence organic inflammatory responses through the modulation of pro-inflammatory and anti-inflammatory cytokines (Lee et al.) (4). This opens up new perspectives on pain management, especially in situations where muscular inflammation plays a key role in pathogenesis.

Within the various issues related to the musculoskeletal system, common problems can affect various body regions, such as the lower back, neck, upper limb (primarily shoulders and elbows but also hand problems), and lower limb (including knees and feet).

The aim of this study is to evaluate how acupuncture may alter the perception of pain experienced by patients, differentiating the results according to the various body regions for which it is used.

## 2 Methods

The fundamental data on which this research is based were extracted from a database that took shape at the Acupuncture clinic managed by the Local Health Authority (ASL) in the city of Turin. This database gradually developed over a prolonged period of over 14 years, covering a time frame from 2008 to 2022.

The Acupuncture clinic played a central role in collecting and accumulating data from a wide spectrum of patients and treatments. The database not only contains patients' clinical progress over the years but also details the various treatment modalities used by different professionals who have worked there over the years.

### 2.1 Patient selection

The database was created by aggregating information from patients who underwent treatment at our clinic. These data were collected both during the initial clinical encounter and in conjunction with the last treatment session. Access to the clinic was strictly by recommendation from the General Practitioner or a specialist, with no direct patient access. In the outpatient clinic, and consequently in the study, all patients referred for musculoskeletal pain were treated, regardless of the cause and intensity of the pain. Patients treated for internal medical conditions were excluded from the study.

In the context of our study, only data from patients who voluntarily agreed to share this information at the beginning and at the conclusion of their treatment were included in our dataset. Our focus was on evaluating the Numeric Rating Scale (NRS) parameter, as expressed by patients initially before the first appointment and at the end of treatment. This parameter represents a key element for measuring pain intensity and symptomatology, providing valuable information about the improvement achieved during treatment.

To analyze and process the collected data, we used JASP software version 0.17.2.1.

### 2.2 Procedure

After the initial encounter, during which the patient's reported issues were thoroughly explored to formulate a personalized treatment plan, the treatment began on the same day. An initial cycle of six sessions, with weekly frequency, was scheduled for each patient. In each subsequent appointment, the patient was asked to provide feedback on the session, and based on the response, the previously selected points were either confirmed or modified. At the end of this cycle, if the physician believed that further improvements were possible, an additional four sessions were scheduled to optimize the results.

Each acupuncture session was conducted by qualified medical professionals with experience in the field. These treatments were based on millennia-old Traditional Chinese Medicine techniques, ensuring a comprehensive and targeted approach to each patient's individual needs. Over weeks, the patient was allowed to use analgesics as needed, exactly as they were doing before initiating the treatment; however, no additional medications were prescribed.

## 3 Results

Between 2008 and 2022, a study was conducted on a total of 1,186 patients. Of these, 932 were treated for musculoskeletal conditions, while 254 had internal medical conditions and were excluded from the research discussed here. The sampled population consisted of individuals aged 23–94, divided into 242 males and 690 females. The patient's characteristics are summarized in Table 1.

**Table 1:** Patient age range and sex.

Sex	Number	Minimum age	Maximum age	Mean
Males	242	28	88	65
Females	690	23	94	66
Total	932	23	94	66

On 932 patients treated, 582 were deemed sufficient with a session count of 6 by the evaluating physician at the conclusion of the sixth session (62.5% of total cases), while for the remaining 350 (37.5%), additional 4 sessions were deemed beneficial and conducted, resulting in a total of 10 sessions.

For this dataset, the average NRS value before and after treatment was 7.49 (±1.69 SD or standard deviation) and 4.27 (±2.06 SD), respectively, with an average reduction in the NRS score of 3.22, indicating a 43% decrease in perceived pain. Given the non-normal distribution of the sampled population, the Wilcoxon test was used to assess the statistical significance of the observed difference, which was confirmed with *p* < 0.001.

When dividing the cases by specific conditions, patients reporting lumbar and cervical symptoms numbered 425 and 232, respectively, making them the largest subgroups in the study. Patients reporting hip pain numbered 19, knee pain 62, foot pain 20, shoulder pain 71, elbow pain 59, and hand pain 44. For each of these categories, the previously described process was repeated.

Among the 425 patients with lumbar problems, 398 had NRS values both at the beginning and at the end of treatment. The values were 7.47 (±1.67 SD) and 4.46 (±2.06 SD), respectively, with an average decrease of 3.01 or 40%. The population exhibited a non-normal distribution, and the result was significant with *p* < 0.001 (Wilcoxon test).

Among the 232 patients with cervical problems, 217 had NRS values both at the beginning and at the end of treatment. The values were 7.41 (±1.73 SD) and 4.24 (±1.97 SD), respectively, with an average decrease of 3.17 or 43%. The population exhibited a non-normal distribution, and the result was significant with *p* < 0.001 (Wilcoxon test).

Among the 19 patients with hip problems, 18 had NRS values both at the beginning and at the end of treatment. The values were 7.89 (±1.38 SD) and 4.59 (±2.16 SD), respectively, with an average decrease of 3.3 or 42%. The population exhibited a normal distribution, so the paired T-test was used, resulting in significance with *p* < 0.001.

Among the 62 patients with knee problems, 56 had NRS values both at the beginning and at the end of treatment. The values were 7.53 (±1.74 SD) and 4.38 (±2.16 SD), respectively, with an average decrease of 3.15 or 42%. The population exhibited a non-normal distribution, and the result was significant with *p* < 0.001 (Wilcoxon test).

Among the 20 patients with foot problems, 18 had NRS values both at the beginning and at the end of treatment. The values were 7.33 (±1.37 SD) and 4.11 (±2.22 SD), respectively, with an average decrease of 3.22 or 44%. The population exhibited a normal distribution, so the paired T-test was used, resulting in significance with *p* < 0.001.

Among the 71 patients with shoulder problems, 66 had NRS values both at the beginning and at the end of treatment. The values were 7.9 (1.8 SD) and 4.29 (2.04 SD), respectively, with an average decrease of 3.61 or 46%. The population exhibited a non-normal distribution, and the result was significant with *p* < 0.001 (Wilcoxon test).

Among the 59 patients with elbow problems, 58 had NRS values both at the beginning and at the end of treatment. The values were 7.44 (±1.37 SD) and 3.37 (±1.95 SD), respectively, with an average decrease of 4.07 or 55%. The population exhibited a normal distribution, so the paired *T*-test was used, resulting in significance with *p* < 0.001.

Among the 44 patients with hand problems, 36 had NRS values both at the beginning and at the end of treatment. The values were 7.1 (±2.07 SD) and 3.5 (±2.21 SD), respectively, with an average decrease of 3.6 or 51%. The population exhibited a non-normal distribution, and the result was significant with *p* < 0.001 (Wilcoxon test).

The results are summarized in Table 2.

**Table 2:** Summary of results no. S (sample size)—V.S. (valid samples)—Diff. (difference between initial NRS and final NRS).

District	No. S	C.V.	Initial NRS	Finale NRS	Diff.	% improvement	*p*
Lumbar	425	398	7.47 (±1.67 SD)	4.46 (±2.06 SD)	3.01	40%	<0.001
Cervical	232	217	7.41 (±1.73 SD)	4.24 (±1.97 SD)	3.17	43%	<0.001
Hip	19	18	7.89 (±1.38 SD)	4.59 (±2.16 SD)	3.3	42%	<0.001
Knee	62	56	7.53 (±1.74 SD)	4.38 (±2.16 SD)	3.15	42%	<0.001
Foot	20	18	7.33 (±1.37 SD)	4.11 (±2.22 SD)	3.22	44%	<0.001
Shoulder	71	66	7.9 (±1.8 SD)	4.29 (±2.04 SD)	3.61	46%	<0.001
Elbow	59	58	7.44 (±1.37 SD)	3.37 (±1.95 SD)	4.07	55%	<0.001
Hand	44	36	7.1 (±2.07 SD)	3.5 (±2.21 SD)	3.6	51%	<0.001
Total	932	867	7.49 (±1.67 SD)	4.27 (±2.06 SD)	3.22	43%	

Subsequently, we assessed whether the differences in improvements among different conditions were random or statistically significant. Initially, the Kruskal–Wallis test was used, which showed a statistically significant difference (*p* 0.009), and then the Dunn test was used, with the results reported in Table 3.

**Table 3:** Significance level of the difference in treatment efficacy among various body segments.

District	*p*
Lumbar—cervical	0.386
Lumbar—knee	0.988
Lumbar—elbow	<.001***
Lumbar—hip	0.596
Lumbar—shoulder	0.023*
Lumbar—foot	0.626
Lumbar—hand	0.199
Cervical—knee	0.635
Cervical—elbow	0.002**
Cervical—hip	0.824
Cervical—shoulder	0.103
Cervical—foot	0.857
Cervical—hand	0.403
Knee—elbow	0.005**
Knee—hip	0.643
Knee—shoulder	0.099
Knee—foot	0.670
Knee—hand	0.300
Elbow—hip	0.135
Elbow—shoulder	0.203
Elbow—foot	0.125
Elbow—hand	0.147
Hip—shoulder	0.512
Hip—foot	0.975
Hip—hand	0.740
Shoulder—foot	0.487
Shoulder—hand	0.704
Foot—hand	0.713

* *p* < .05.

** *p* < .01.

*** *p* < .001.

The Kruskal–Wallis test was also applied using gender and age decade as dependent variables, but it yielded inconclusive levels of significance (*p* 0.5 and 0.15, respectively).

## 4 Discussion

The conducted investigation involved a large cohort of over 1,000 patients, with long-term monitoring spanning over 14 years. Through an in-depth analysis of the collected data, it emerged that acupuncture is a highly effective method for treating musculoskeletal pain, as assessed by the Numeric Rating Scale (NRS). The results reveal a remarkable average reduction in initial pain, amounting to 43%.

A significant aspect is that acupuncture demonstrates a statistically significant reduction in pain in all the considered body regions, with varying percentages depending on the anatomical location. These values range from 40% for lumbar pain to 55% for elbow pain, highlighting the extensive beneficial scope of the technique.

In detail, the analysis reveals that acupuncture can contribute to an average decrease of 40% in lumbar pain, 42% for hip joint pathologies and knee pain, 43% for cervical pain, 44% for foot pain, 46% for shoulder pain, 51% for hand pain, and 55% for elbow pain. It is interesting to note that the data suggest that acupuncture treatment appears to be particularly effective for elbow-related issues, showing a reduction of over 50% in initial suffering. Furthermore, a greater treatment effectiveness is observed in the shoulder region compared to the lumbar regions. However, no differences were observed in the treatment of male or female patients or among patients of different ages. Nevertheless, it is crucial to underline that in our study, the sample size varies considerably depending on the different anatomical regions examined, potentially introducing an initial bias that requires further investigation, especially in cases which have been less represented in the present study.

The importance of these results paves the way for further research in the field, potentially outlining more targeted and optimized therapeutic approaches for each body region. In conclusion, acupuncture emerges as a valid therapeutic option for musculoskeletal pain treatment, offering significant benefits that warrant further exploration through larger and diversified studies, without being burdened by the weight of significant side effects (only mild and transient hematomas were reported during the study).

## Data Availability

The original contributions presented in the study are included in the article/Supplementary Material, further inquiries can be directed to the corresponding author.
